# Retained portion of the appendix following laparosocpic appendicectomy causing peritonitis and ileus

**DOI:** 10.4103/0972-9941.78353

**Published:** 2011

**Authors:** B P Gouda, R T Kochar, R S Shah

**Affiliations:** Department of Surgery, P.D. Hinduja National Hospital and Medical Research Centre, Veer Savarkar Marg, Mahim, Mumbai-400007, India; 1 Department of Pediatric Surgeon, P.D. Hinduja National Hospital and Medical Research Centre, Veer Savarkar Marg, Mahim, Mumbai-400007, India

**Keywords:** Laparoscopy, complication, appendicectomy, acute appendicitis

## Abstract

We describe a patient who developed peritonitis and paralytic ileus due to a retained portion of the inflammed appendix following laparoscopic appendicectomy (LA). The details of the presentation and management are discussed along with a brief review of the unusual complications LA.

## INTRODUCTION

Laparoscopic appendicectomy (LA) has become the standard of care in children with acute appendicitis (AA). A recent meta-analysis showed a significantly reduced incidence of wound infection of 1.5% in LA compared with 5% in open appendicectomy (OA).[1] Despite some early apprehensions regarding a higher incidence of intra-abdominal abscesses following LA; the same report indicated that the frequency of this complication was comparable following LA and OA. We report here an unusual case of peritonitis and paralytic ileus in the immediate postoperative period following LA due to a retained appendicular tip.

## CASE REPORT

An 11-year-old boy was brought to us 4 days after having undergone a LA for AA at a peripheral hospital. The surgery had been undertaken some 48 hours after the onset of symptoms and an inflammed appendix had been removed. His pain had resolved after surgery, only to return after 24 hours. He was unable to tolerate oral liquids and had developed loose motions from the second postoperative day onwards. In view of his unsatisfactory progress, he was brought to our tertiary care hospital.

On arrival, he was in severe pain with a pulse of 116/ min, blood pressure of 100/84 mm Hg and respiratory rate of 24/minute. His abdomen showed generalised distension and tenderness along with rigidity. The bowel sounds were diminished. His investigations revealed a haemoglobin of 11.6 gm% and white cell count of 16,200/mm^3^ with 80% neutrophils. The serum electrolytes and serum creatinine were normal. An erect chest X-ray showed free gas under the diaphragm and abdominal X-ray showed multiple air fluid levels with ground glass appearance in pelvis. Computed tomography (CT) revealed dilatation of small bowel loops with thickening of one of the loops. Free fluid along with air pockets was seen in the peritoneal cavity. An emergent laparotomy was undertaken through a right supra-umbilical transverse incision. This revealed presence of faeculent fluid in the peritoneal cavity and dilated bowel loops. The appendicular stump, caecum and colon were unremarkable. Thorough examination of the bowel failed to reveal any perforation. A tubular structure measuring 3-cm in length was found floating freely between distended small bowel loops; this was retrieved. A through lavage was given and a drain was placed in pelvis. Postoperatively the boy made a slow but satisfactory recovery and was discharged on day 7. A superficial wound infection he developed was treated by laying the wound partially open and dressing it. Histopathological examination of the retrieved tubular structure was confirmed to be a part of the appendix. At the follow-up visit at 8 weeks he had recovered completely.

## DISCUSSION

Unusual complications following LA have been reported in the surgical literature. Duron *et al*.[2] in a multi-centric study found 0.16% incidence of postoperative intestinal obstruction following LA. Rarely, postoperative caecal[3] or midgut volvulus[4] in the absence of congenital malrotation have also been described. Use of stapler for excising the appendix is not uncommon in the West and small bowel obstruction due to staples lying loose in the abdominal cavity has been reported by at least a couple of authors.[5,6] In the early days after introduction of LA, concerns were raised regarding possibility of “residual” appendicitis as a result of incomplete appendicectomy.[7] It was stressed that the junction between the appendix and the caecum should be identified unequivocally before controlling and excising the appendix. Rare instances of a residual appendix becoming adherent to the terminal ileal mesentery and causing intestinal obstruction due to internal herniation are known, as reported by Gordon *et al*.[8]

Guillem *et al*. and Geoghegan and colleagues have alluded to the risk of development of intra-abdominal abscesses following LA due to an appendicolith dropping into the peritoneal cavity.[9,10] Both authors strongly advocate a systematic division of the appendix between two ligatures. Also a watchful eye for a faecolith during division of the appendix and its prompt retrieval should obviate this complication. In our patient it was the retained portion of the appendix, which presumably fell back into the abdomen during retrieval that acted as a nidus of infection and led to the peritonitis and paralytic ileus. Although the precise etiology was not suspected preoperatively, the clinical and imaging findings prompted us to undertake an early laparotomy and this lead to a rapid resolution of the condition and a satisfactory outcome.

Our case helps underscore some important tenets that need to be followed during LA. The details of the method of delivery of the specimen during the first surgery in our case were not available. In the presence of a non-inflammed, relatively thin appendix it is usually possible to retrieve the specimen through the 10-mm umbilical port after changing over to a 5-mm laparoscope that is shifted to one of the ancilliary ports. Alternately, as previously reported by us the appendix can be retrogradely introduced into the 10-mm port and removed.[11] However, utmost care should be taken that the specimen is retrieved in its entirety, as friable appendices are liable to breakage during extraction. Our preference in presence of a bulky inflammed appendix is to place it in a plastic bag and retrieve it through the umbilical port – if necessary after slightly enlarging the fascial incision. This “non-contact” technique not only ensures removal of the entire specimen but, despite the absence of any objective data in support, this practice should also reduce the risk of port-site infection.

In conclusion, during LA careful dissection of the entire appendix, secure control of its base, avoidance of spillage of faecoliths or a portion of the appendix during surgery and, when necessary, retrieval of a bulky specimen in a plastic bag should help avoid such unusual infective complications.

## References

[1] Aziz O, Athanasiou T, Tekkis PP, Purkayastha S, Haddow J, Malinovski V (2006). Laparoscopic versus open appendectomy in children a meta-analysis. Ann Surg.

[2] Duron JJ, Hay JM, Msika S, Gaschard D, Domergue J, Gainant A (2000). Prevalence and mechanisms of small intestinal obstruction following laparoscopic abdominal surgery: A retrospective multicenter study. French Association for Surgical Research. Arch Surg.

[3] McIntosh SA, Ravichandran D, Wilmink AB, Baker A, Purushotham AD (2001). Cecal volvulus occurring after laparoscopic appendectomy. JSLS.

[4] Cuadra SA, Khalife ME, Char DJ, Wax MR, Halpern D (2002). Intestinal obstruction from midgut volvulus after LA. Surg Endosc.

[5] Nottingham JM (2002). Mechanical small bowel obstruction from a loose linear cutter staple after LA. Surg Laparosc Endosc Percutan Tech.

[6] Huntington TR, Klomp GR (1995). Retained staples as a cause of mechanical small-bowel obstruction. Surg Endosc.

[7] Marcoen S, Onghena T, Van Loon C, Vereecken L (1999). Residual appendicitis following incomplete laparoscopic appendectomy. Acta Chir Belg.

[8] Gordon R, Bamehriz F, Birch DW (2004). Residual appendix producing small-bowel obstruction after LA. Can J Surg.

[9] Guillem P, Mulliez E, Proye C, Pattou F (2004). Retained appendicolith after Laparoscopic Appendectomy: The need for systematic double ligature of the appendiceal base. Surg Endosc.

[10] Geoghegan T, Stunnell H, O’Riordan J, Torreggiani WC (2004). Retained appendicolith after laparoscopic appendectomy. Surg Endosc.

[11] Bhandarkar D, Shah R (2002). A novel technique for extraction of the appendix in laparoscopic appendectomy. Surg Laparosc Endosc Percutan Tech.

